# The Correlation Between Lateral Ventricle Asymmetry and Cerebral Blood Flow: Implications for Stroke Risk

**DOI:** 10.3390/diagnostics15243126

**Published:** 2025-12-08

**Authors:** Xiaojia Sun, Wenjie Gao, Shanshan Gao, Xudong Wang, Honglin Feng

**Affiliations:** Department of Neurology, First Affiliated Hospital of Harbin Medical University, No. 23 Youzheng Street, Nangang District, Harbin 150001, China

**Keywords:** cerebral blood flow (CBF), computed tomography (CT), lateral ventricle volume, cerebral artery stenosis, stroke

## Abstract

**Background:** This study explored the correlation between lateral ventricle volume asymmetry and cerebral blood flow (CBF). **Methods:** A retrospective review of 94 patients who underwent CT perfusion (CTP) and standard brain imaging was conducted. Lateral ventricle volumes and CBF across 13 vascular-based regions of interest (ROIs) were measured. Cerebral artery stenosis was identified using magnetic resonance angiography (MRA) and digital subtraction angiography (DSA). Paired *t*-tests, Pearson’s correlation, logistic regression, and Cox models were used to assess the relationships between lateral ventricle asymmetry, CBF differences, and their associations with cerebral artery stenosis and the risk of stroke during follow-up. **Results:** 94 patients were included (mean age: 60.7 years). CBF was significantly lower on the side of the larger lateral ventricle in regions supplied by the anterior cerebral artery (ACA) (Mean relative value ± SD, % = 112.3 ± 32.5, *p*-value = 0.0016) and middle cerebral artery (MCA) (Mean relative value ± SD, % = 123.1 ± 57.8, *p*-value = 0.0004). A moderate correlation was observed between lateral ventricle volume asymmetry and CBF differences across the entire cohort. Significant associations were identified between CBF differences in specific ROIs and the presence of cerebral artery stenosis (MCA: aOR = 1.026, 95% CI: 1.004–1.048, *p*-value = 0.019). **Conclusions:** Lateral ventricle asymmetry is associated with reduced CBF in specific brain regions, particularly those supplied by the ACA. CBF differences in regions supplied by the PCA are linked to increased risk of subsequent stroke during follow-up.

## 1. Introduction

Cardiovascular disease (CVD)—which includes coronary heart disease, cerebrovascular disease, and peripheral vascular disease—represents a major global public health concern and remains the leading cause of mortality in China [1,2]. Stroke, a primary manifestation of CVD, ranks as the fifth leading cause of mortality in the U.S. [3], highlighting the critical need for early screening and timely intervention in high-risk individuals. Previous studies have reported that stenosis of more than 50% in a vessel lumen is likely to cause a mild stroke [4,5,6]. Stenosis can lead to transient or definite neurological symptoms, or it may remain asymptomatic, depending on the severity, reversibility of ischemia, and the presence of collateral circulation. The mechanisms of cerebral infarction secondary to intracranial artery stenosis include large artery-to-artery embolism, hemodynamic compromise, local branch occlusion, and in situ thrombosis [7,8,9]. Hemodynamic changes resulting from cerebral artery stenosis, such as chronic cerebral hypoperfusion, can significantly increase the risk of stroke [10]. Possible mechanisms include reduced blood flow on small thrombi [11], or reduced cerebral vascular autoregulation function [12]. Stenotic cerebral arteries heighten cerebrovascular activity, and the compensatory adjustments increase stroke risk [13]. Accordingly, identifying cerebral artery stenosis before a stroke occurs is crucial for effective stroke prevention.

Lateral ventricle asymmetry is observed in approximately 5–12% of individuals and is often considered a normal anatomic variant [14,15]. A study of 170 cases with lateral ventricle asymmetry and an equal number of normal controls found that 3.5% of the asymmetric group had choroidal masses or periventricular dysplasia in further magnetic resonance imaging (MRI) examination [14]. No significant differences were found between the two groups in neurological symptoms, signs, or lifestyle habits, but the study did not assess cerebral artery or perfusion in relation to lateral ventricular asymmetry. Other studies on lateral ventricular asymmetry tend to focus more on investigating its associations with neurodegenerative diseases such as Parkinson’s, epilepsy, dementia, brain trauma, and developmental abnormalities [16,17]. However, in clinical practice, some patients with lateral ventricle asymmetry shown in brain computed tomography (CT) images had no obvious clinical symptoms but had cerebral artery stenosis. We hypothesized that asymptomatic lateral ventricle asymmetry may be associated with abnormal cerebral blood perfusion. Accordingly, this study aimed to investigate whether there is a correlation between lateral ventricle asymmetry, cerebral blood perfusion, and cerebral artery stenosis.

## 2. Methods

### 2.1. Subjects

This retrospective study reviewed the medical records of outpatients who underwent CT perfusion (CTP) in conjunction with standard brain imaging, including CT, MRI, or magnetic resonance angiography (MRA), computed tomography angiography (CTA), or digital subtraction angiography (DSA), at the Department of Neurology in the First Affiliated Hospital of Harbin Medical University from 2017 to 2018. Patients visiting the outpatient clinic generally suffer from chronic dizziness, headache, limb numbness, and pain caused by peripheral neuropathy, insomnia, etc., and seek consultation for previous cerebrovascular diseases. CTP was indicated to assess cerebral hemodynamic status in these patients for suspected cerebral artery stenosis or chronic cerebral blood flow insufficiency based on initial brain imaging. The inclusion period (2017–2018) was selected to ensure a complete 5-year follow-up for all participants, as the follow-up phase was completed in 2023.

Exclusion criteria were patients with one of the following conditions: (1) symptoms of acute cerebral infarction, such as hemiplegia, slurred speech, dizziness lasting more than 1 day, and crooked mouth; (2) acute stroke confirmed by subsequent imaging; (3) a history of cerebral infarction except for lacunar infarction; (4) hydrocephalus; (5) cerebral hemorrhage; (6) craniocerebral trauma; (7) intracranial infection; (8) intracranial mass occupying; (9) congenital craniocerebral malformation; (10) acute neurological symptoms.

Follow-up: Five years after the examinations, subjects’ survival and stroke occurrence were tracked through telephone interviews or medical record reviews. Stroke events were defined as new-onset ischemic stroke, intracerebral hemorrhage (ICH), or spontaneous subarachnoid hemorrhage (SAH) occurring after the baseline imaging assessment and diagnosed by a physician. Transient ischemic attacks (TIA) were not included as stroke events. Ischemic stroke was defined as a new focal neurological deficit lasting ≥24 h with confirmation on CT or MRI, or as an acute ischemic infarction documented by the treating physician in the electronic medical record. Hemorrhagic stroke (ICH or SAH) was defined as a spontaneous intracranial hemorrhage confirmed by neuroimaging or recorded by the treating physician.

During follow-up, incident stroke events were identified through structured telephone interviews and a review of electronic medical records:

(1) For participants who were re-admitted to our hospital during the follow-up period, stroke status was determined by reviewing electronic medical records according to the diagnostic criteria described above, as confirmed by review of the electronic medical records, including discharge summaries and neuroimaging reports.
(2) For participants who did not return to our hospital, stroke status was determined through structured telephone interviews. A stroke event was recorded only when the participant or a family member confirmed that the event had been diagnosed by a licensed physician and that the episode required emergency evaluation or hospitalization.

Follow-up was noninvasive and limited to telephone interviews and review of electronic medical records. No additional imaging, laboratory testing, or interventions were performed as part of the protocol. The study protocol was reviewed and approved by the Ethics Committee of the First Affiliated Hospital of Harbin Medical University, and determined that written informed consent was not required due to the retrospective design and the use of de-identified data. All procedures were conducted in accordance with the ethical standards of the institutional and national research committees, as well as the 1964 Declaration of Helsinki and its subsequent amendments.

### 2.2. Determination of the Lateral Ventricle Volume of Both Sides

The outlines of the bilateral ventricles of each subject were manually drawn on each cross-sectional image, and the corresponding area (mm^2^) of each slice was measured. Multiple measurements were performed to minimize errors. The scanning thickness was 5 mm, and the height of each ventricle was estimated by multiplying the thickness of each slice by the number of slices. The volume of each lateral ventricle was calculated by summing the products of the outlined areas and the corresponding slice thickness [18].

All participants underwent imaging to assess bilateral ventricle volumes. In each case, the volume of the lateral ventricle on the larger side was designated as the “larger lateral ventricle volume (LLV)”, while the volume on the smaller side was termed the “smaller lateral ventricle volume (SLV)”. Because lateral ventricle volumes were analyzed as continuous measures (rather than classifying ventricles as ‘symmetric’ or ‘asymmetric’ by a cutoff), we did not calculate a specific prevalence of ventricular asymmetry in the cohort.

### 2.3. Determination of CBF

CT scans were conducted utilizing Philips Brilliance 16 CT scanner (Philips, Amsterdam, The Netherlands). CBF data were derived through Philips Extended Brilliance Workspace (EBW) platform (version 4.5) with the brain perfusion module, which applies the central volume principle (CBF (mL/100 g/min) = cerebral blood volume (CBV)/mean transit time (MTT)) [19]. Subsequently, the difference in CBF between the two lateral ventricles was quantified using the ratio of their respective CBF values (CBF_SLV_/CBF_LLV_) × 100%, adopting a self-control approach. This difference was expressed as relative CBF. Values > 100% indicate that CBF is higher on the SLV side (i.e., relatively lower on the LLV side).

Based on the distributions of intracranial blood vessels, the area surrounding the lateral ventricles was divided into specific regions of interest (ROI). These ROIs were delineated using the image processing workstation on brain CT. Referring to the Alberta Stroke Program Early CT Score and the posterior circulation Acute Stroke Prognosis Early CT score [20,21], the area around the lateral ventricle was selected. Through appropriate merging, addition and organization, 13 ROIs were determined as follows (Supplementary Figure S1):

ROI 1: cortex area supplied by anterior cerebral artery (ACA) (frontal and parietal lobe);

ROI 2: cortex area supplied by middle cerebral artery (MCA) (frontal and parietal lobe);

ROI 3: cortex area supplied by ACA (frontal lobe);

ROI 4: cortex area supplied by MCA (temporal lobe);

ROI 5: cortex area supplied by posterior cerebral artery (PCA);

ROI 6: cortex area supplied by ACA (frontal lobe);

ROI 7: cortex area supplied by MCA (temporal lobe);

ROI 8: deep area supplied by MCA (including the caudate nucleus, the lenticular nucleus, and the internal capsule);

ROI 9: deep area supplied by PCA (thalamic);

ROI 10: cortex area supplied by PCA (occipital lobe);

ROI 11: the area supplied by ACA (uncinate gyrus);

ROI 12: cortex area supplied by MCA (temporal lobe);

ROI 13: cortex area supplied by PCA (occipital lobe).

Accordingly, the corresponding relative CBF values for each ROI were obtained through CTP. Note that some ROI labels (e.g., MCA temporal, PCA occipital) appear more than once in the list; this is because we sampled those vascular territories at distinct, non-overlapping axial levels, not that the same layer was duplicated.

### 2.4. Ascertainment of Cerebral Artery Stenosis

MRA was performed in all participants as the standard noninvasive method to assess arterial stenosis, acquired on either a GE Optima MR360 1.5 T or a GE Signa HDxt 3.0 T system (GE Healthcare, Chicago, IL, USA). CTA was conducted only in patients whose initial noninvasive screening—such as MRA, transcranial Doppler (TCD), or carotid ultrasound—revealed obvious stenosis or when clinicians needed to exclude high-risk vascular conditions.

DSA was performed only in a subset of patients who consented to further interventional evaluation and potential stent placement as part of their clinical management. All DSA procedures were conducted using a Philips Allura Xper system (Philips Medical Systems Nederland BV, Eindhoven, The Netherlands) by experienced neuroradiologists. DSA findings were primarily utilized to confirm MRA-detected lesions and to guide subsequent therapeutic decisions. To minimize subjective variability, all image evaluations were performed by the radiologists using multiple visual assessments.

### 2.5. Statistical Analysis

Descriptive statistics for the study subjects are presented as counts (n) and percentages (%) for categorical data and as the mean ± standard deviation (SD) for continuous data. Since this study used each subject’s lateral ventricles as internal controls to explore whether the CBF on the side of the LLV is lower than that on the side of the SLV, a paired sample *t*-test was conducted. An independent *t*-test with Bonferroni correction was conducted to compare the differences in relative CBF across various ROIs between patients with and without unilateral stenosis in the anterior circulation. Repeated-measures ANOVA was conducted when analyses involved all 13 ROIs simultaneously to account for within-subject correlations.

Pearson’s correlation coefficients were calculated to evaluate the relationship between lateral ventricle volume asymmetry (i.e., the difference in volume between the LLV and SLV) and the corresponding relative CBF. Univariate logistic regression analyses were performed to assess the association between the presence of intracranial stenosis (dependent variable: any stenosis vs. none) and each of the following independent variables: the lateral ventricular volume difference and the relative CBF for each ROI. Cox proportional hazards models were used to evaluate whether ventricular volume asymmetry or relative CBF in each ROI was associated with time to stroke during follow-up. Each ROI’s relative CBF was tested in a separate model adjusting for age, sex, and unilateral stenosis, yielding adjusted hazard ratios (aHRs) for stroke. ROI-level logistic and Cox analyses were considered exploratory, and *p*-values are unadjusted for multiplicity.

To evaluate the consistency between the side of the LLV and the side of unilateral stenosis in patients with anterior circulation stenosis, Cohen’s Kappa (κ) was calculated. This statistical measure assesses inter-rater agreement for categorical variables, accounting for agreement expected by chance.

Statistical analyses were performed using IBM SPSS software, version 22.0 (IBM Corp., Armonk, NY, USA). All tests were two-tailed, and a *p*-value less than 0.05 was considered statistically significant.

## 3. Results

### 3.1. Patients’ Characteristics

**Figure 1:**
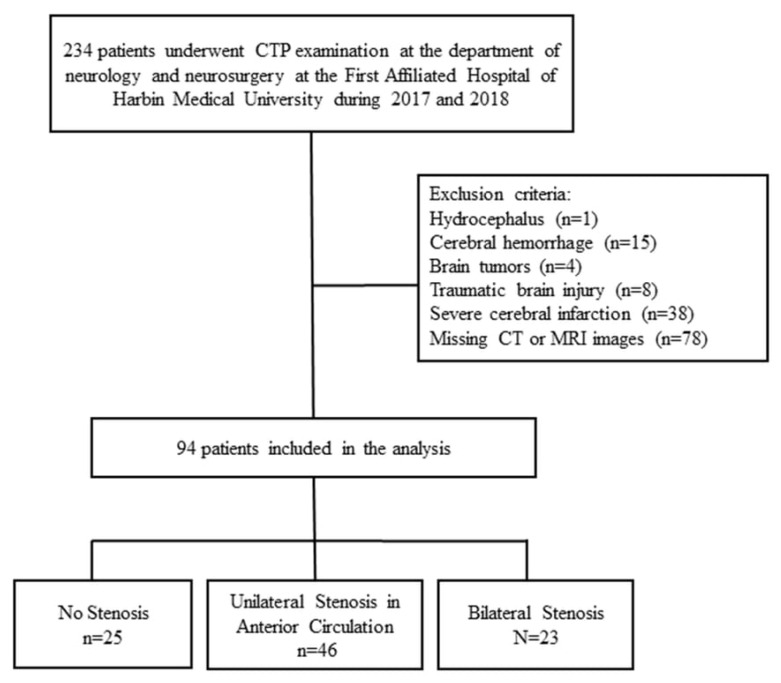
Flow chart of the subject selection.

The flowchart of subject selection is presented in Figure 1. A total of 94 participants were included in the analysis. The characteristics of the included patients are summarized in Table 1. The mean age was 60.7 years, with the majority being male (63.8%). The majority of individuals had a larger ventricle on the left side (58.5%). In addition, 46 patients (48.9%) had unilateral stenosis (in the anterior circulation), 23 patients (24.5%) had bilateral stenosis, and 25 (26.6%) had no evidence of stenosis. At 5 years after entry, 12 (12.8%) patients died, and 22 (23.4%) experienced strokes (Table 1).

### 3.2. Comparison Between CBF on the Side of the LLV and the Side of the SLV Across the ROIs

Table 2 presents the comparison of CBF between the side of the LLV and the side of the SLV across the ROIs, expressed as relative CBF values (CBF_SLV_/CBF_LLV_, in percentage). Thus, relative CBF values above 100% consistently reflected lower perfusion on the LLV side. Significantly higher absolute CBF (mL/100 g/min) was observed in the SLV compared to the LLV in regions supplied by the ACA (ROIs 1, 3, and 6) and the MCA (ROIs 2, 4, 7, and 12). In all these ROIs, the relative CBF (%) was consistently lower on the LLV side compared to the SLV side (mean relative value > 100%) (Table 2).

### 3.3. Relative CBF Between the LLV and SLV Sides Across the ROIs in Patients with and Without Unilateral Stenosis

**Figure 2:**
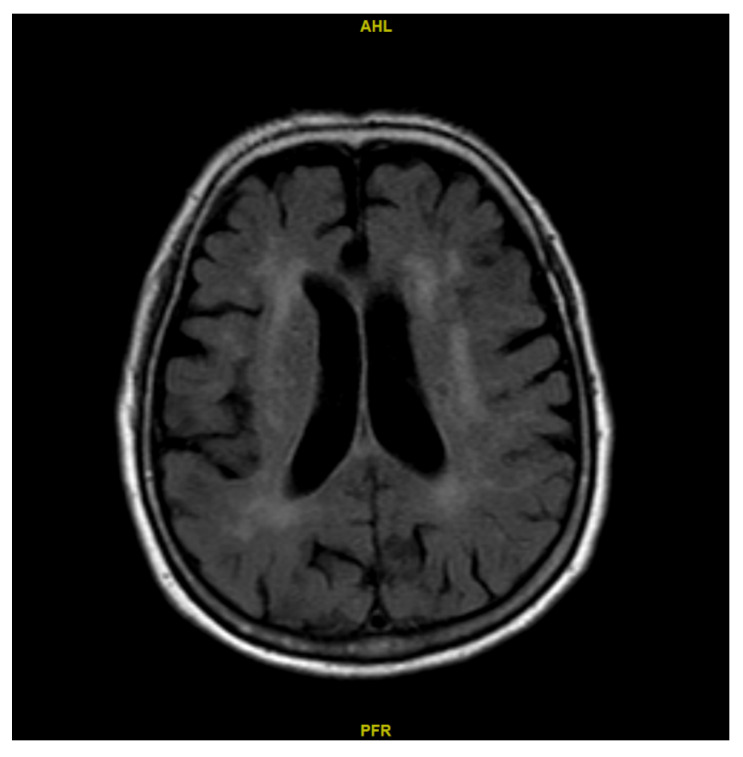
Axial MRI (T2-weighted FLAIR) showing left–right ventricular asymmetry and mild periventricular white matter hyperintensity.

**Figure 3:**
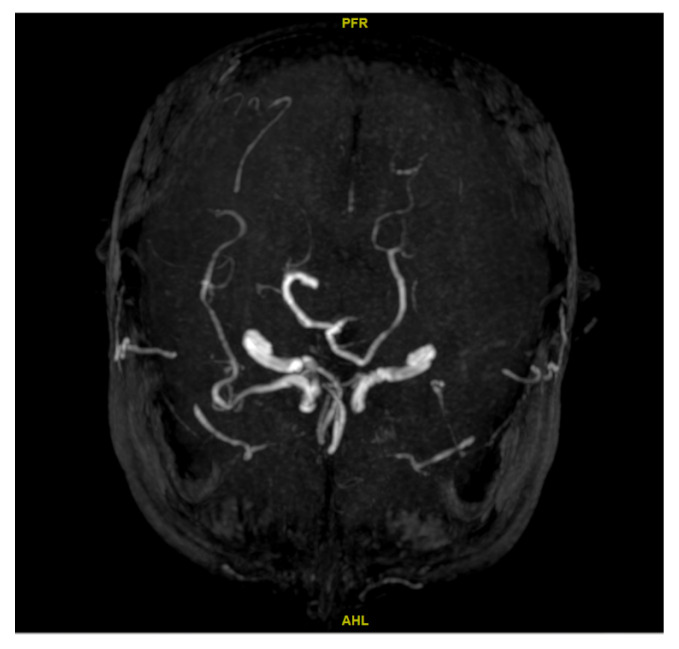
3D time-of-flight MRA demonstrating left MCA occlusion.

Subsequently, we stratified patients by the presence of unilateral anterior-circulation stenosis to examine CBF differences. Supplementary Table S1 shows the relative CBF between the LLV and SLV sides across the ROIs in patients with and without unilateral stenosis. Among the patients who had unilateral stenosis, significant differences (*p* < 0.05) in relative CBF between the LLV and SLV were observed mainly in ACA and MCA regions. In addition, relative CBF was significantly greater among patients with unilateral stenosis compared to the others in ROI 2 (relative CBF, without unilateral stenosis: 116.1% vs. with unilateral stenosis: 152.3%, *p* = 0.022) (Supplementary Table S1). We further investigated the relationship between lateral ventricle volume and artery stenosis (Figure 2 and Figure 3). There was a strong agreement between the side of stenosis and the side with the LLV among 46 subjects presented with unilateral stenosis, as indicated by a κ value of 0.803 (Supplementary Table S2).

Parameters: TR = 7000 ms, TE = 125 ms, TI = 2300 ms, slice thickness = 6 mm, FOV = 230 × 230 mm.

Parameters: TR = 20 ms, TE = 3.5 ms, flip angle = 18°, slice thickness = 2.0 mm, FOV = 160 × 160 mm.

### 3.4. Correlation Between the Lateral Ventricle Volume Difference and Relative CBF Across the ROIs

Table 3 shows the correlation between the lateral ventricle volume difference and the relative CBF across various ROI. For the entire patient group (n = 94), significant moderate positive correlations were observed in ROI 3 (r = 0.247, *p* < 0.05) and ROI 6 (r = 0.212, *p* < 0.05), both located in the territory of the ACA. When stratifying the patients based on the presence of unilateral stenosis, the subgroup without unilateral stenosis (n = 48) showed a significant correlation in ROI 3 (r = 0.331, *p* < 0.05). In contrast, the subgroup with unilateral stenosis (n = 46) did not exhibit any significant correlations in any of the ROIs, although moderate correlation coefficients were noted in ROI 1 (r = 0.245) and ROI 6 (r = 0.283).

### 3.5. Associations Between the Lateral Ventricle Volume Difference, Relative CBF Across ROIs, and Presence of Stenosis

Table 4 presents the associations between lateral ventricle volume differences, relative CBF across ROIs, and the presence of stenosis, as determined by univariate logistic analyses. Significant associations were observed between relative CBF and the presence of any stenosis compared to no stenosis in ROI 2 (OR: 1.03, 95% CI: 1.006–1.062, *p* = 0.018), ROI 4 (OR: 1.02, 95% CI: 1.001–1.042, *p* = 0.039), and ROI 7 (OR: 1.02, 95% CI: 1.000–1.033, *p* = 0.050). No significant association was found between relative CBF and the presence of unilateral stenosis across all ROIs. Notably, the magnitude of ventricular volume asymmetry itself was not significantly associated with stenosis presence in these analyses (*p* > 0.05).

### 3.6. Associations Between the Lateral Ventricle Volume Difference, Relative CBF Across ROIs, and Stroke During Follow-Up

Table 5 presents the associations between lateral ventricle volume differences, relative CBF across ROIs, and the occurrence of any stroke events during follow-up. Significant associations were observed between relative CBF in the PCA regions and the incidence of stroke, specifically in ROI 10 (adjusted HR: 1.007, 95% CI: 1.001–1.014, *p* = 0.022) and ROI 13 (adjusted HR: 1.013, 95% CI: 1.004–1.022, *p* = 0.003). No significant associations were found in other ROIs or with lateral ventricle volume differences. No significant association was found between the absolute ventricular volume difference and stroke occurrence (*p* > 0.1).

## 4. Discussion

In summary, our study revealed that patients consistently had lower CBF on the side of the larger lateral ventricle across multiple brain regions, particularly in areas supplied by the ACA and MCA. We also found a positive correlation between the degree of ventricular volume asymmetry and relative CBF differences in ACA territories, although this correlation was modest. Additionally, greater interhemispheric CBF disparities in certain regions (notably in MCA territories) were associated with the presence of intracranial arterial stenosis. Importantly, perfusion differences in PCA-supplied regions (occipital cortex) were linked to an increased risk of stroke during follow-up.

Overall, this study highlights the clinical significance of assessing lateral ventricular asymmetry and the associated CBF differences, particularly in relation to cerebral artery stenosis and stroke risk, with a focus on asymptomatic patients. The findings suggest that relative CBF, especially in specific cerebral regions, may serve as important indicators for early intervention and stroke prevention in patients with asymptomatic lateral ventricular asymmetry. While the association between cerebral artery stenosis and reduced cerebral perfusion has been previously studied [10], this study provides new clinical data supporting this hypothesis through specific CBF measurements and comparative analysis of lateral ventricular volumes. Our findings underscore the clinical significance of incorporating CTP in the evaluation of patients with incidental ventricular asymmetry, as it may unmask hemodynamic abnormalities that would otherwise go undetected.

As mentioned earlier, prior studies on lateral ventricle differences primarily focused on neurodegenerative diseases, trauma, and developmental abnormalities [16,17]. This study investigated the relationship between lateral ventricle volume differences and cerebral blood perfusion in 94 participants. We found that in the anterior circulation supply area, CBF is significantly greater on the side with the smaller lateral ventricle. This aligns with the side of stenosis in unilateral anterior circulation arterial narrowing, consistent with the larger ventricle side. In cases of brain damage or stenosis, an increase in intracranial blood volume and pressure can lead to ventricular enlargement, a phenomenon well documented as a radiologic hallmark after traumatic brain injury [22,23]. Our findings align with this mechanism, suggesting that chronic hemodynamic stress may similarly contribute to ventricular dilation in asymptomatic patients.

This study also observed that among participants with existing arterial stenosis, numerous brain regions exhibited significant bilateral differences in blood flow. This is consistent with the expected hemodynamic effect of arterial stenosis, where multiple brain regions can show bilateral perfusion differences. However, correlation analysis indicates that only a subset of brain regions demonstrates statistically significant associations on relative CBF with arterial narrowing, and the correlation is not strong. Regression analysis yields similar results. The circle of Willis includes the ACA, MCA, and PCA, helping maintain perfusion [24,25]. The cerebral arteries are interconnected via the circle of Willis, allowing blood flow redistribution between anterior and posterior circulations and between hemispheres. If one segment is stenosed, collateral flow from other vessels can often preserve perfusion enough to avoid ischemic symptoms [26]. Thus, the blood flow rate can be maintained in a reasonable range with little difference between sides. Additionally, intracranial arteries can undergo compensatory enlargement in response to atherosclerotic plaque formation, which helps sustain blood flow and further attenuates measurable perfusion asymmetry until stenosis becomes critical [27]. Collectively, these mechanisms likely attenuate measurable interhemispheric asymmetry in many ROIs despite the presence of stenosis.

Localized blood flow changes typically precede visible arterial stenosis in imaging, explaining the relatively low concordance between cerebral blood flow changes and imaging-detected stenosis in this study [19,28]. A prior study has proposed that posterior cerebral arteries exhibit thinner walls, reduced elastin content, and more concentric intima thickening compared to anterior cerebral arteries [29]. In contrast, PCA end-zone cortex in the occipital lobes (ROIs 10 and 13) combines greater interindividual anatomic variability (e.g., fetal-type PCA) with generally weaker posterior-circulation collateral capacity and the high metabolic demand of the visual cortex, rendering these regions less tolerant to flow reductions [30,31,32]. These characteristics may explain why PCA-territory perfusion asymmetry tracked with subsequent stroke in our cohort. Given the exploratory nature of these analyses, this observation should be regarded as hypothesis-generating and requires confirmation in future prospective studies.

Our cohort reflects targeted outpatient use of CTP—typically prompted by duplex ultrasound or MRA suggesting focal stenosis—rather than population screening. This design increases clinical relevance for high-risk patients but limits generalizability; conclusions should therefore not be extrapolated to asymptomatic community samples.

In this study, bilateral lateral ventricle differences show no statistically significant correlation with arterial stenosis or stroke occurrence. While the asymmetry of lateral ventricles can be considered a normal variation in brain structure, it also holds the potential to serve as a predictor for intracranial pathology or cognitive disorders, particularly among the elderly population. Anatomically, the periventricular region is characterized by a high concentration of white matter [33]. Damage to neural axons, demyelination, and disruptions in connections between white matter fibers can lead to a direct reduction in brain tissue volume or, indirectly, cause cortical damage by impeding the upward transmission of energy due to white matter degeneration [34,35]. Previous investigations have established associations between asymmetrical lateral ventricles and motor asymmetry in conditions such as Parkinson’s disease [16], Alzheimer’s disease [36], and the presence of moderate to severe white matter lesions [37,38]. Moreover, pronounced asymmetry in lateral ventricles can result in midline shift, ventricle compression, and elevation of intracranial pressure [39,40]. In such instances, compromised blood flow accompanies the progression of brain atrophy [41]. In other words, the regulation of brain blood flow constitutes an autoregulated system, independent of stroke or stenosis. The findings of this study align with and substantiate the concept of autoregulation within the brain’s circulatory dynamics.

We hypothesized a causal chain wherein regional cerebral hypoperfusion—rather than ventricular asymmetry itself—plays a central upstream role that may precede angiographically visible arterial stenosis and ultimately contribute to stroke risk. Our data partially support this: we observed a strong association between ventricular asymmetry and reduced CBF, and in patients with stenosis, the larger ventricle corresponded to the stenotic side. However, we did not find ventricular asymmetry to be a significant predictor of stenosis presence or future stroke. One explanation is that our imaging assessments captured mainly large-artery stenoses; subtler small-vessel disease, which can cause brain atrophy and ventricular enlargement [42], would not be detected on MRA/DSA. It is known that microvascular lesions can reduce cerebral blood flow in gray or white matter and contribute to brain atrophy [37,43]. Therefore, ventricular asymmetry in our patients might be driven by microvascular ischemic changes that precede any large artery stenosis visible on imaging. Alternatively, ventricular enlargement and perfusion changes may occur earlier in the pathologic process, whereas detectable arterial stenosis and stroke occur later. In this scenario, by the time a stroke or a severe stenosis develops, the initial ventricular changes might not show a direct correlation, as they happened well before the endpoint.

Our cohort included high-risk outpatients who underwent CTP after duplex ultrasound or MRA indicated focal stenosis, making our findings most relevant to this population rather than to the general community. Consequently, our conclusions should not be generalized to asymptomatic samples, and potential selection bias may exist because CTP is often ordered for patients with more complex or uncertain vascular findings. Future studies should employ broader screening strategies and include more diverse patient groups to evaluate the role of CTP in different clinical settings. For example, arterial spin labeling (ASL) may serve as a noninvasive, contrast-free MRI technique for quantifying cerebral blood flow, offering a safer alternative for longitudinal assessment of cerebral hemodynamics [44]. Taken together, these findings support a pragmatic, targeted workflow: if pronounced ventricular asymmetry is incidentally identified, a focused CBF assessment may be warranted to prioritize patients for advanced hemodynamic evaluation while avoiding indiscriminate CTP. In this context, CTA-based measures such as the cortical venous opacification score (COVES) may provide an intermediate layer of assessment to refine patient selection before proceeding to CTP [45,46]. In unilateral large-vessel stenosis, asymmetry–CBF correlations were modest and non-significant, warranting cautious interpretation. Prospective studies are needed to determine whether such a triage strategy improves outcomes and to validate whether PCA end-zone perfusion asymmetry (ROIs 10/13) provides incremental prognostic information.

### Strength and Limitations

The study had several limitations. First, a small sample size at a single center may have selection bias and limit the generalization of results to other populations or locations. As this was a retrospective study, some clinical variables were incomplete and therefore not included in the multivariable analysis. Future studies with more comprehensive data collection are warranted to establish a more complete predictive model and to further clarify the relationships. Second, the causal relationship between lateral ventricle volume enlargement and blood flow reduction remains unclear, as the sequence of these events is unknown in our investigation. Third, manual measurement of ventricular volume may introduce variability or measurement bias. Fourth, because TOF-MRA relies on flow-related enhancement, it may overestimate high-grade stenosis in areas of turbulent or slow flow and has lower sensitivity for mild intracranial atherosclerosis when compared with contrast-enhanced MRA or DSA. Additionally, our CTP analysis provided relative CBF values rather than absolute quantification. Although this approach may introduce measurement error, we attempted to mitigate it by using within-subject comparisons (ratio of bilateral CBF). Although quantitative CBF values from CT perfusion may have inherent measurement errors, we attempted to mitigate this limitation by comparing relative CBF between the bilateral ventricles and employing a self-control approach. Lastly, the severity of arterial stenosis was not classified in detail; stenosis was categorized only as present or absent, without further stratification based on severity, which may have limited the precision of our analysis. Further approaches, such as computational modeling and automated data analysis, should be employed to enhance measurement accuracy.

## 5. Conclusions

This study highlights the clinical significance of assessing lateral ventricle asymmetry and corresponding relative CBF, particularly in relation to cerebral artery stenosis and stroke risk. The findings suggest that, although causal links have yet to be confirmed, lateral ventricle asymmetry is associated with reduced CBF in specific brain regions, particularly those supplied by the ACA. Additionally, relative CBF in regions supplied by the PCA were significantly associated with an increased risk of stroke. These results underscore the importance of incorporating CBF analysis into the evaluation of patients with asymptomatic lateral ventricle asymmetry to improve early detection and management of cerebrovascular disease. Further research is needed to validate these findings and explore the underlying mechanisms in larger and more diverse populations.

## Figures and Tables

**Table 1:** Characteristics of the patients.

Characteristics	Statistics Mean ± SD or n (%)
Age, years	60.7 ± 9.1
Sex	
Female	34 (36.2)
Male	60 (63.8)
Side of larger ventricle volume	
Left side	55 (58.5)
Right side	39 (41.5)
Presence of cerebral artery stenosis	
No stenosis	25 (26.6)
Unilateral stenosis	46 (48.9)
Bilateral stenosis	23 (24.5)
Follow-up	
Death	12 (12.8)
Stroke	22 (23.4)

**Table 2:** Comparison between CBF on the side of the LLV and the side of the SLV across the ROIs (n = 94).

	SLV Side	LLV Side	Paired Difference ^a,b^	
	Mean ± SD (mL/100 g/min)		Relative CBF, Mean ± SD, %	*p*-Value
**Lateral ventricle volume, mm^3^**	37,109.7 ± 28,289.8	48,292.8 ± 34,112.3		
**CBF relative value, %**	72.5 ± 33.4	66.7 ± 29.8	110.2 ± 26.6	0.0004 *
**ACA**	68.4 ± 33.2	62.7 ± 31.1	112.3 ± 32.5	0.0016 *
ROI 1	63.1 ± 36.6	55.0 ± 32.1	124.6 ± 56.1	0.0026 *
ROI 3	66.2 ± 37.5	56.7 ± 29.8	120.0 ± 40.9	<0.001 *
ROI 6	67.0 ± 35.8	61.3 ± 31.2	113.3 ± 42.3	**0.0315**
ROI 11	77.3 ± 42.6	77.8 ± 44.2	108.3 ± 47.4	0.8723
**MCA**	76.3 ± 37.4	65.7 ± 30.1	123.1 ± 57.8	0.0004 *
ROI 2	70.1 ± 37.5	59.3 ± 30.0	133.8 ± 102.7	0.0010 *
ROI 4	64.9 ± 34.6	55.5 ± 28.9	131.5 ± 85.3	0.0026 *
ROI 7	94.4 ± 81.9	74.5 ± 35.3	142.2 ± 126.2	**0.0142**
ROI 8	83.8 ± 42.6	80.6 ± 43.2	110.8 ± 49.2	0.3908
ROI 12	68.2 ± 38.6	58.4 ± 31.9	132.3 ± 123.8	0.0002 *
**PCA**	71.9 ± 35.2	72.0 ± 34.6	100.9 ± 16.1	0.9190
ROI 5	65.1 ± 33.8	63.3 ± 38.3	110.1 ± 34.4	0.3558
ROI 9	82.3 ± 44.3	81.5 ± 41.7	102.6 ± 24.0	0.7420
ROI 10	71.1 ± 37.9	72.9 ± 37.7	102.6 ± 41.5	0.4179
ROI 13	69.2 ± 36.1	70.3 ± 37.1	101.5 ± 30.5	0.5376

^a^ Absolute CBF values are expressed in mL/100 g/min. relative CBF values are expressed as percentages. a Paired differences in lateral ventricle volume: LLV-SLV. ^b^ Paired differences in CBF across ROIs: CBFSLV-CBFLLV. *p*-values < 0.05 are shown in bold. * *p*-values < 0.0038 (Bonferroni corrected significant level). Repeated-measures ANOVA *p* = 0.0004. CBF, cerebral blood flow; ROI, region of interest; SLV, smaller lateral ventricle; LLV, larger lateral ventricle; SD, standard deviation.

**Table 3:** Correlations between the lateral ventricle volume difference and CBF relative value (%) between LLV and SLV sides across ROIs.

Pearson’s Correlation Coefficient, r	With Unilateral Stenosis		
	All (n = 94)	Yes (n = 46)	No (n = 48)
**ACA**	0.182	0.194	0.178
ROI 1	0.162	0.245	0.136
ROI 3	0.247 *	0.266	0.331 *
ROI 6	0.212 *	0.283	0.182
ROI 11	−0.046	−0.106	−0.042
**MCA**	−0.069	−0.079	−0.052
ROI 2	−0.032	−0.086	0.077
ROI 4	0.011	−0.049	0.105
ROI 7	−0.116	−0.098	−0.136
ROI 8	0.014	0.015	0.032
ROI 12	−0.056	0.055	−0.083
**PCA**	−0.010	−0.279	0.116
ROI 5	0.181	0.078	0.273
ROI 9	−0.043	−0.212	0.060
ROI 10	0.136	−0.138	0.207
ROI 13	−0.023	−0.182	0.023

CBF, cerebral blood flow; ROI, region of interest; SLV, smaller lateral ventricle; LLV, larger lateral ventricle. Significance are shown in bold: * *p* < 0.05. Lateral ventricle volume difference = LLV − SLV. CBF difference = CBF_SLV_ − CBF_LLV_.

**Table 4:** Associations between the lateral ventricle volume difference, CBF relative value between LLV and SLV sides across ROIs, and presence of stenosis.

	Any Stenosis		Unilateral Stenosis	
	OR	*p*-Value	OR	*p*-Value
**Lateral ventricle volume difference, mm^3^**	1.00 (0.999, 1.00)	0.647	1.00 (0.999, 1.000)	0.209
**CBF relative value, %**	1.030 (0.999, 1.061)	0.055	1.004 (0.989, 1.020)	0.604
**ACA**	1.011 (0.994, 1.029)	0.213	0.998 (0.986, 1.011)	0.773
ROI 1	1.003 (0.994, 1.012)	0.499	1.001 (0.994, 1.008)	0.770
ROI 3	1.010 (0.996, 1.024)	0.149	1.007 (0.996, 1.017)	0.207
ROI 6	1.006 (0.992, 1.021)	0.369	0.998 (0.989, 1.008)	0.750
ROI 11	1.003 (0.993, 1.014)	0.552	0.995 (0.986, 1.004)	0.323
**MCA**	1.026 (1.004, 1.048)	**0.019**	1.004 (0.996, 1.011)	0.352
ROI 2	1.022 (1.002, 1.042)	**0.029**	1.005 (0.999, 1.011)	0.126
ROI 4	1.014 (1.001, 1.027)	**0.038**	1.003 (0.998, 1.008)	0.302
ROI 7	1.015 (1.000, 1.029)	**0.046**	0.999 (0.995, 1.002)	0.473
ROI 8	1.015 (0.996, 1.034)	0.113	1.003 (0.994, 1.011)	0.547
ROI 12	0.999 (0.995, 1.002)	0.460	0.999 (0.996, 1.003)	0.719
**PCA**	1.00 (0.97, 1.03)	0.922	1.004 (0.979, 1.030)	0.749
ROI 5	1.00 (0.99, 1.01)	0.966	1.002 (0.990, 1.014)	0.726
ROI 9	1.00 (0.99, 1.02)	0.648	1.005 (0.988, 1.023)	0.540
ROI 10	1.00 (0.99, 1.01)	0.906	0.998 (0.987, 1.008)	0.641
ROI 13	1.01 (0.99, 1.03)	0.468	0.998 (0.984, 1.011)	0.736

Lateral ventricle volume difference = LLV − SLV. CBF, cerebral blood flow; ROI, region of interest; SLV, smaller lateral ventricle; LLV, larger lateral ventricle. Significance (*p* < 0.05) are shown in bold.

**Table 5:** Associations between the lateral ventricle volume difference, CBF relative value between LLV and SLV sides across ROIs, and any stroke during follow-up.

	Crude HR (95% CI)	*p*-Value	Adjusted HR (95% CI) ^a^	*p*-Value
**Lateral ventricle volume difference, mm^3^**	1.00 (0.999, 1.00)	0.679	1.00 (0.999, 1.000)	0.574
**CBF relative value, %**	1.004 (0.990–1.017)	0.609	1.005 (0.991–1.019)	0.516
**ACA**	1.003 (0.991–1.015)	0.610	1.003 (0.991–1.015)	0.621
ROI 1	0.997 (0.989–1.006)	0.536	0.998 (0.989–1.006)	0.579
ROI 3	1.000 (0.989–1.010)	0.970	1.001 (0.990–1.012)	0.850
ROI 6	1.006 (0.997–1.014)	0.182	1.006 (0.998–1.014)	0.175
ROI 11	1.003 (0.995–1.010)	0.447	1.002 (0.995–1.010)	0.568
**MCA**	0.999 (0.992–1.007)	0.851	1.000 (0.992–1.008)	0.969
ROI 2	0.999 (0.994–1.004)	0.716	1.000 (0.995–1.004)	0.921
ROI 4	0.999 (0.993–1.004)	0.623	0.999 (0.993–1.005)	0.750
ROI 7	0.998 (0.993–1.003)	0.456	0.998 (0.993–1.003)	0.415
ROI 8	1.000 (0.992–1.008)	0.975	1.000 (0.992–1.009)	0.914
ROI 12	1.000 (0.998–1.003)	0.775	1.000 (0.998–1.003)	0.854
**PCA**	1.028 (1.008–1.049)	**0.005**	1.029 (1.010–1.049)	**0.003**
ROI 5	1.007 (0.996–1.017)	0.209	1.008 (0.997–1.019)	0.142
ROI 9	1.001 (0.984–1.018)	0.906	1.001 (0.985–1.018)	0.862
ROI 10	1.008 (1.001–1.014)	**0.017**	1.007 (1.001–1.014)	**0.022**
ROI 13	1.014 (1.005–1.022)	**0.003**	1.013 (1.004–1.022)	**0.003**

^a^ Multivariable analysis adjusted for age, sex, and unilateral stenosis. HR, hazard ratio; CBF, cerebral blood flow; ROI, region of interest; SLV, smaller lateral ventricle; LLV, larger lateral ventricle. Significance (*p* < 0.05) are shown in bold.

## Data Availability

The data sets used and/or analyzed during the current study are available from the corresponding author on reasonable request.

## Supplementary Materials

The following supporting information can be downloaded at: https://www.mdpi.com/article/10.3390/diagnostics15243126/s1, Table S1: Relative CBF value between the LLV and SLV sides across ROIs in patients with and without unilateral stenosis.; Table S2: Consistency between the LLV side and the stenosis side in patients with unilateral stenosis in the anterior circulation.

## Abbreviations

ROI, region of interest; CT, computed tomography; MRI, magnetic resonance imaging; MRA, magnetic resonance angiography.
