# Supplementary material for: The Correlation Between Lateral Ventricle Asymmetry and Cerebral Blood Flow: Implications for Stroke Risk
Source: Diagnostics (Basel). 2025 Dec 8;15(24):3126. doi: 10.3390/diagnostics15243126 (PMC12731552; doi:10.3390/diagnostics15243126)
Supplement: Supplementary file 1 [file diagnostics-15-03126-s001.zip › diagnostics-3873191-supplementary.pdf]

Supplementary Table S1. relative CBF value between the LLV and SLV sides across ROIs in patients with and without unilateral stenosis.

	Unilateral stenosis				
	No		Yes		
**CBF relative value, %**	**Mean relative value ± SD, %**	**p-value [a]**	**Mean relative value ± SD, %**	**p-value [a]**	**p-value for between-group difference [b]**
**ACA**	113.2 ± 34.8	**0.0192**	111.3 ± 30.2	**0.0348**	0.3388
ROI 1	122.9 ± 59.5	0.1031	126.3 ± 52.9	**0.0114**	0.4248
ROI 3	114.7 ± 31.9	**0.0155**	125.5 ± 48.4	**0.0020***	**0.0052**
ROI 6	114.6 ± 49.1	0.1202	111.8 ± 34.4	0.1437	**0.0183**
ROI 11	113.0 ± 52.0	0.2971	103.3 ± 41.9	0.2890	0.1488
**MCA**	117.6 ± 44.6	**0.0147**	128.9 ± 68.9	**0.0113**	**0.0037***
ROI 2	116.1 ± 61.3	0.2589	152.3 ± 131.1	**0.0016***	**<0.0010***
ROI 4	122.3 ± 60.3	0.1287	141.0 ± 105.1	**0.0090**	**0.0002***
ROI 7	151.6 ± 158.7	0.0668	132.4 ± 80.2	**0.0397**	**<0.0010***
ROI 8	107.8 ± 40.7	0.2280	113.9 ± 57.1	0.7222	**0.0229**
ROI 12	136.9 ± 166.5	**0.0295**	127.6 ± 51.8	**0.0020***	**<0.0010***
**PCA**	100.4 ± 17.7	0.9907	101.4 ± 14.4	0.8891	0.1697
ROI 5	108.9 ± 32.2	0.5588	111.4 ± 36.9	0.4776	0.3542
ROI 9	101.1 ± 23.9	0.8818	104.1 ± 24.2	0.6060	0.9232
ROI 10	104.5 ± 51.9	0.9367	100.5 ± 26.9	0.2808	**<0.0010***
ROI 13	102.5 ± 36.9	0.8025	100.4 ± 22.2	0.5433	**0.0008***

[a] The paired sample t-test of CBF between LLV and SLV sides.
[b] The independent t-test of CBF relative value between with and without unilateral stenosis.
P-values <0.05 are shown in bold.
*P-values < 0.0038 (Bonferroni corrected significant level)
Repeated ANOVA p-value of CBF between LLV and SLV sides in patients with unilateral stenosis = 0.009
Repeated ANOVA p-value of CBF between LLV and SLV sides in patients without unilateral stenosis = 0.017
Repeated ANOVA p-value of CBF relative value between with and without unilateral stenosis = 0.523
CBF, cerebral blood flow; ROI, region of interest; SD, standard deviation.

Supplementary Table S2. Consistency between the LLV side and the stenosis side in patients with unilateral stenosis in the anterior circulation.

LLV side	Stenosis side		Sum
	Left	Right	
**Left**	29 (63.0%)	3 (6.5%)	32
**Right**	1 (2.2%)	13 (28.3%)	14
**Sum**	30	16	46 (100%)

Kappa=0.803
LLV, larger lateral ventricle
